# Image-driven modeling of the proliferation and necrosis of glioblastoma multiforme

**DOI:** 10.1186/s12976-017-0056-7

**Published:** 2017-05-02

**Authors:** Vishal Patel, Leith Hathout

**Affiliations:** 1 Department of Radiological Sciences Ronald Reagan-UCLA Medical Center, University of California, Los Angeles, 757 Westwood Plaza, Suite 1638, Los Angeles, 90095 CA USA; 2 Harvard Medical School, 25 Shattuck Street, Boston, 02115 MA USA

**Keywords:** Glioblastoma, Magnetic resonance imaging, Diffusion tensor imaging, Computational modeling

## Abstract

**Background:**

The heterogeneity of response to treatment in patients with glioblastoma multiforme suggests that the optimal therapeutic approach incorporates an individualized assessment of expected lesion progression. In this work, we develop a novel computational model for the proliferation and necrosis of glioblastoma multiforme.

**Methods:**

The model parameters are selected based on the magnetic resonance imaging features of each tumor, and the proposed technique accounts for intrinsic cell division, tumor cell migration along white matter tracts, as well as central tumor necrosis. As a validation of this approach, tumor growth is simulated in the brain of a healthy adult volunteer using parameters derived from the imaging of a patient with glioblastoma multiforme. A mutual information metric is calculated between the simulated tumor profile and observed tumor.

**Results:**

The tumor progression profile generated by the proposed model is compared with those produced by existing models and with the actual observed tumor progression. Both qualitative and quantitative analyses show that the model introduced in this work replicates the observed progression of glioblastoma more accurately relative to prior techniques.

**Conclusions:**

This image-driven model generates improved tumor progression profiles and may contribute to the development of more reliable prognostic estimates in patients with glioblastoma multiforme.

## Background

Glioblastoma multiforme (GBM), the most common primary brain neoplasm in adults, represents the most malignant end of the glioma spectrum. Though the prognosis is generally poor, there is considerable variability in response to treatment and patient outcomes. This variation suggests that the optimal therapeutic approach likely incorporates an individualized assessment of expected tumor progression. To that end, there has been considerable interest in developing computational models that accurately replicate the observed temporal evolution of GBM lesions.

The most widely-studied models for GBM progression take the form of reaction-diffusion systems, a well-characterized class of partial differential equations [1–4]. In the context of predicting GBM evolution, reaction-diffusion models of the form represented by Eq. 1 describe the tumor cell concentration within the affected tissue as a function of space and time:

1 $$  \frac{\partial c\left(\mathbf{x},t\right)}{\partial t} = Q\left(\mathbf{x},t\right) + R\left(\mathbf{x},t\right)  $$

The core underlying principle postulates that the change in tumor cell concentration (*c*) at a particular location (**x**) over time (*t*) is driven by: 1) a diffusion term (*Q*(**x**,*t*)), accounting for the net flux of tumor cells from adjacent locations, and 2) a proliferation term (*R*(**x**,*t*)), representing the intrinsic cell multiplication rate.

Initial models considered tumor cell diffusivity (*D*) to be spatially and temporally invariant (i.e., *Q*(**x**,*t*)=*D*∇^2^ *c*), producing spherical profiles of tumor progression [1, 4–7]. Such models were later refined to allow for observed differences in diffusivity within gray and white matter, yielding *Q*(**x**,*t*)=∇·(*D*(**x**)∇*c*), with *D*(**x**) taking on one of two values, depending on the underlying tissue type at **x** [8]. While this change addressed the differential rates of GBM progression through gray and white matter, it did not accurately reproduce the propensity for GBM to invade along white matter tracts [9–12]. Ultimately, the incorporation of the voxelwise diffusion tensor, **D**(**x**), allowed for the successful simulation of preferential tumor cell migration along fiber bundles [13]:

2 $$  Q\left(\mathbf{x},t\right) = \nabla \cdot \left(\mathbf{D}\left(\mathbf{x} \right)\nabla c \right)  $$

Regarding the proliferation term, a variety of models for cell multiplication have been previously described, including exponential growth (*R*(**x**,*t*)=*ρ* *c*), Gompertz growth (*R*(**x**,*t*)=*ρ* *c* ln(*c*_*m*_/*c*)), and logistic growth (*R*(**x**,*t*)=*ρ* *c*(1−*c*/*c*_*m*_)), where the parameter *ρ* controls the growth rate and *c*_*m*_ represents the maximum cell concentration [14]. Each of these functions generates monotonically increasing tumor cell densities, albeit with differing temporal dynamics. GBM, however, is an aggressive malignancy that commonly outstrips the supporting capacity of its underlying substrate resulting in central necrosis, a property that has been neglected by the existing proliferation models [15–17]. If, however, computational models are to serve as adjuncts for clinical decision-making, accurate simulation of this tumoral necrosis is of critical importance.

The purpose of the present work is to develop a computational model that is both driven by the observed imaging characteristics of each individual tumor and also based on the most accurate descriptions of GBM cell diffusion and proliferation, including central necrosis. In the following sections, we introduce our proposed computational construct, outline its practical implementation, and qualitatively and quantitatively evaluate its performance relative to existing techniques.

## Methods

### Model construction

We propose a model that combines the diffusion tensor driven migration of cells along white matter bundles (Eq. 2) with logistic tumor cell proliferation and with a novel necrosis term which is activated once cell density has surpassed tissue supporting capacity:

3 $$  \frac{\partial c\left(\mathbf{x},t\right)}{\partial t} = \left\{ \begin{array}{lr} \nabla \cdot \left(\mathbf{D}\left(\mathbf{x} \right)\nabla c \right) + \rho c \left(1-{c}/{c_{m}}\right) & : \max \left[c\left(\mathbf{x}\right)\right] < \tau \\ \eta c & : \max \left[c\left(\mathbf{x}\right)\right] \geq \tau \end{array} \right.  $$

We introduce the necrosis rate, *η*∈(0,1), which produces an exponential decay of tumor cells once cell concentration exceeds a threshold, *τ*. Here, max[*c*(**x**)] denotes the maximum cell concentration at position **x** across time; in keeping with empiric observations, our model presumes a physiologic change that maintains the necrotic state even as cell death causes the tumor cell density to fall below *τ*.

**Fig. 1:**
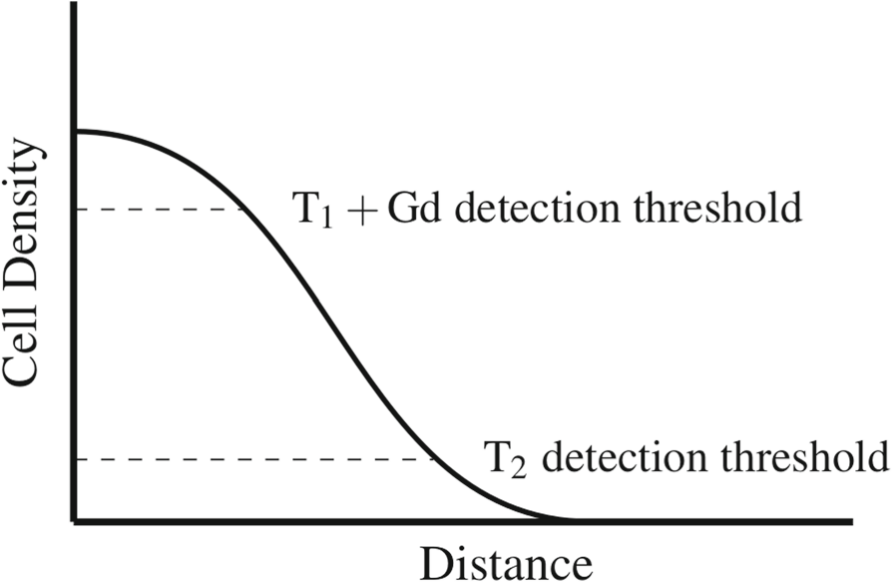
Estimation of GBM growth parameters. Tumor radii measured on serial MR imaging studies were used to calculate tumor-specific model parameters. The T_2_-weighted and contrast-enhanced T_1_-weighted tumor radii correspond to 16 and 80% of the maximal cell density, respectively

We utilize MR imaging features of each GBM lesion to estimate model parameters, thus producing a customized, tumor-specific predictor of future growth and proliferation. Specifically, we measure tumor radii on serial T_2_-weighted and contrast-enhanced T_1_-weighted images, which have been hypothesized to correspond to cell densities of 0.16*c*_*m*_ and 0.80*c*_*m*_, respectively [18, 19]. This relationship is illustrated schematically in Fig. 1. Given the tumor radial velocity (*v*) and cell density gradients thus estimated, Fisher’s relationship ($v=2\sqrt []{\rho D}$) enables the calculation of the proliferation parameter, *ρ*, and cell diffusivity, *D* [5]. We note the physiological implications of this choice of sequences. The enhancing tumor corresponds to areas of angioneogenesis with capillary leak. The T_2_ margin represents infiltrating nonenhancing tumor and associated vasogenic edema; the notion of subthreshold tumor beyond the area of T_2_ abnormality has also been described and is important in radiation therapy planning [20].

As has been previously described, to account for the observed strong preferential migration of GBM along white matter tracts, we consider **D** as the tumor cell diffusion tensor, derived via a differential scaling of the eigenvalues of the symmetric, positive definite spin diffusion tensor calculated from diffusion-weighted MRI [13, 21]. Furthermore, we scale **D** such that the mean cell diffusivity matches the *D* estimated from the serial MR images as described above.

We fix *c*_*m*_=10^5^cells/mm^3^ as in previous work [13]. The necrosis threshold, *τ*, is chosen as a fraction of *c*_*m*_, and is customized for each tumor based upon the earliest detection of necrosis on contrast-enhanced T_1_-weighted imaging; lesions which exhibit earlier necrosis have lower values for *τ*. Finally, *η* is also empirically selected for each tumor based on the width of the enhancing rim on T_1_-weighted imaging such that lesions with narrower regions of enhancement have lower *η* (more rapid necrosis).

### Model validation

To validate our model, we simulate tumor growth in the brain of a healthy adult volunteer using parameters derived from the imaging data of a rare patient with GBM and serial preoperative imaging. We computationally seed tumor cells in a voxel within the brain of our volunteer corresponding to the location of the observed tumor isocenter, and we study the simulated GBM progression over time. Allowing for intersubject anatomical variability—which we attempt to minimize by using an age and gender matched volunteer—we expect that an ideal model would reproduce the observed tumor growth very closely. Thus, the ability of our simulation to recreate the lesion provides insight into the validity of our technique. We qualitatively compare the observed tumor profile to those produced by the proposed and prior models. We further perform a quantitative comparison by nonlinearly registering the two individuals’ brains and computing the mutual information between the simulated tumor profiles and the contrast-enhanced T_1_-weighted image of the observed tumor. In this context, the mutual information (MI) is a measure that quantifies the amount of information that the simulated cell density shares with the post-contrast T_1_ signal intensity (*s*). If *p*(·) denotes a marginal probability density function and *p*(·,·) indicates a joint probability density function, the mutual information is given by:

4 $$  \text{MI}\left(c;s\right) = \sum_{C \in c} \sum_{S \in s} p\left(C,S\right) \log_{2} \left[\frac{p\left(C,S\right)} {p(C) p (S)} \right]  $$

In addition, to examine the sensitivity of our model to the position of the initial seed voxel, we repeat the above experiment with a fixed *ρ* and *D*, but we displace the initialization voxel in each of four cardinal directions from the original seed. We qualitatively describe the resulting tumor progression profiles in relation to the original, and we also quantify the differences between profiles initialized with slightly different seeds using the mutual information metric as described above.

All images were obtained using a 3 T scanner with typical acquisition parameters for T_1_-weighted (T_R_ = 487 ms, T_E_ = 16 ms, 5 mm slice thickness), T_2_-weighted (T_R_ = 3670 ms, T_E_ = 93 ms, 5 mm slice thickness), and diffusion-weighted (T_R_ = 4100 ms, T_E_ = 95 ms, 4 mm slice thickness) sequences. Diffusion-weighted images were acquired over 64 distinct gradient directions at a *b*-value of 1000mm/s^2^ with 3 repeat acquisitions which were averaged to maximize the signal-to-noise ratio. Following correction for eddy current distortions and head motion, diffusion tensors were estimated from diffusion-weighted MR images using the Diffusion Imaging Reconstruction and Analysis Collection (Laboratory of Neuro Imaging, Los Angeles) [22]. Modeling of tumor progression was performed using custom implementations written in MATLAB®; (MathWorks®;, Natick). Equation 3 was solved using a finite difference scheme with a small discretization timestep (*Δ* *t*=1day) to maintain numerical stability. Registration was performed using the FNIRT utility from FSL (Centre for Functional MRI of the Brain, Oxford) [23].

## Results

**Fig. 2:**
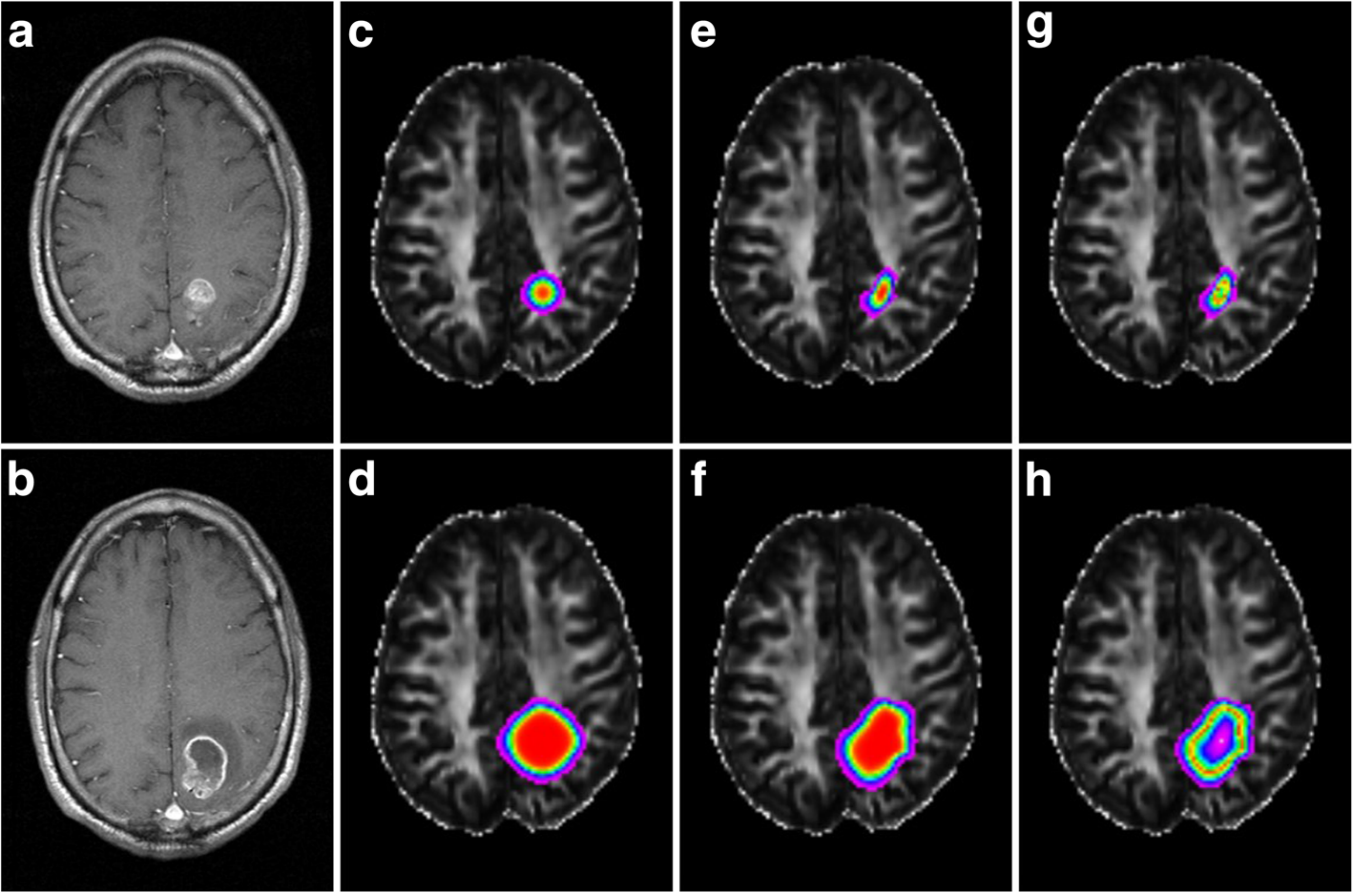
Qualitative comparison of GBM progression models in reproducing tumor progression over 30 days. **a**, **b**: observed tumor; **c**, **d**: isotropic cell diffusion, no necrosis; **e**, **f**: anisotropic cell diffusion, no necrosis; **g**, **h**: anisotropic cell diffusion, with necrosis

Panels A and B of Fig. 2 represent post-contrast T_1_-weighted images, acquired 30 days apart, from an adult male patient who later underwent surgical resection with final histology confirming GBM. For the depicted tumor, we calculated proliferation parameter *ρ*=0.33/day and cell diffusivity *D*=0.0825mm^2^/day. Given that the tumor already exhibits necrosis at the *t*_0_ time point, we found that setting the necrosis threshold just above the T_1_ detection threshold at *τ*=0.85*c*_*m*_ produced comparable behavior. Furthermore, we found that setting *η*=0.9 produced a customized model that replicated the observed thin (2–3 mm) rim of enhancing tumor.

We then attempted, using the proposed and several previously reported models, to reproduce this observed tumor growth profile de novo using anatomical information from MR imaging of a healthy adult volunteer. Panels C – H of Fig. 2 represent simulated tumor evolution using these parameters overlaid upon fractional anisotropy maps derived from our volunteer; red voxels indicate the highest tumor cell concentration, magenta the lowest. Panels C and D display results, over a simulated 30 day interval, from a model that treats tumor cell diffusion as isotropic and does not account for tumor necrosis [2]. Panels E and F display results from a recently-described model that incorporates diffusion anisotropy into tumor cell migration profiles, but does not incorporate a necrosis term [13]. Finally, panels G and H display results over a 30 day interval from a simulation run with our proposed model, which accounts for both anisotropic tumor cell diffusion and central tumoral necrosis. The result produced by Eq. 3 demonstrates a marked qualitative improvement in similarity with the actual tumor growth depicted in panels A and B. In particular, we note that our proposed model is the only one that simulates the thin rim of enhancement and centrally necrotic core observed in the true GBM lesion.

Table 1 summarizes the mutual information between the various models tested in Fig. 2 and the observed tumor at the *t*_0_ and *t*_0_+30day time points. The model allowing only for isotropic cell diffusion and not accounting for necrosis provides the least information about the contrast-enhanced T_1_ signal intensity in the observed tumor. The model allowing for anisotropic cell diffusion but not accounting for necrosis generates a proliferation profile with intermediate mutual information with the true proliferation. Finally, we note that our proposed model produces a tumor profile with the greatest mutual information with the actual tumor proliferation. In addition, we see that for each model, mutual information is greater at the *t*_0_ time point relative to 30 days later.

**Table 1:** Quantitative comparison of GBM progression models

Model	MI (*t*=*t*_0_)	MI (*t*=*t*_0_+30days)
Isotropic cell diffusion, no necrosis	3.1732	2.2624
Anisotropic cell diffusion, no necrosis	3.2107	2.3464
Anisotropic cell diffusion, with necrosis	3.2890	2.4569

The mutual information (MI) between the observed tumor (Fig. 2, panels A and B) and studied models (Fig. 2, panels C – H) is computed at the two available time points

**Fig. 3:**
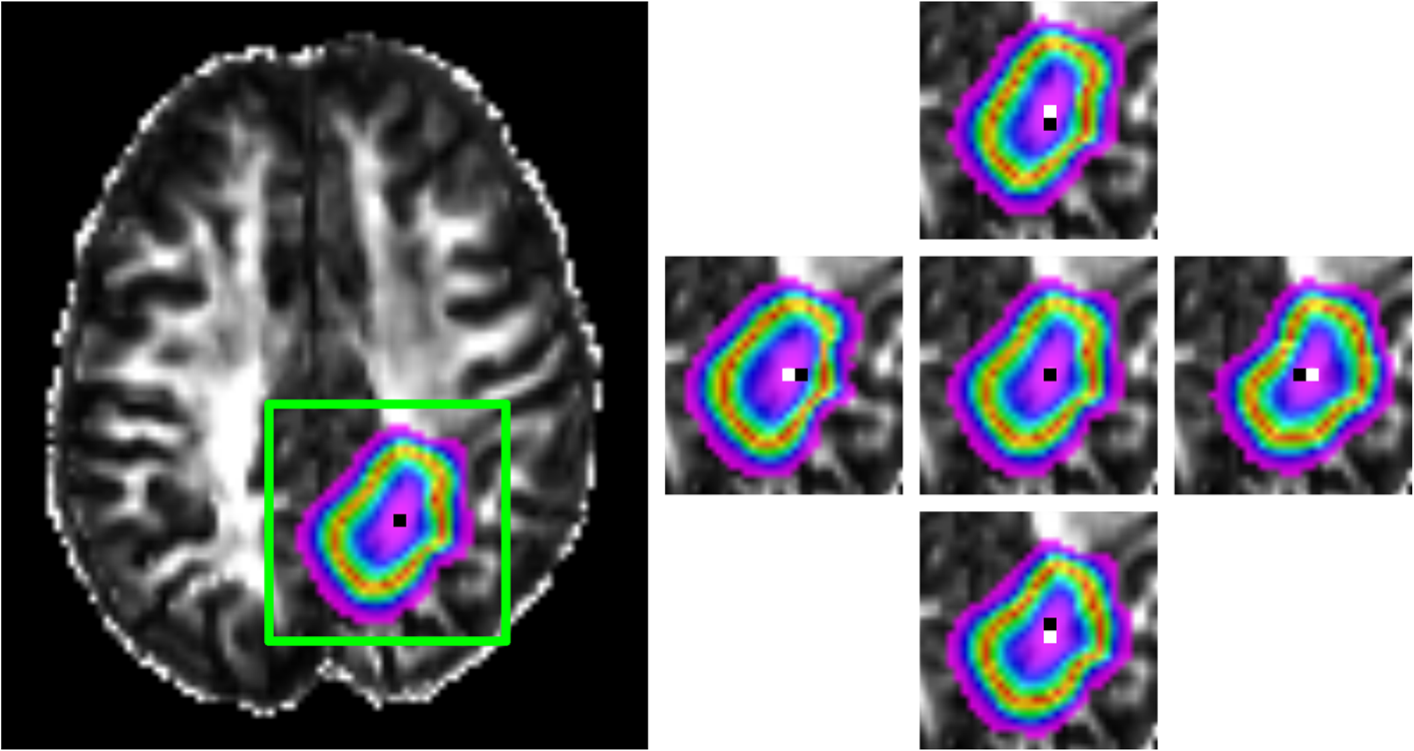
Effects of slight variations in the choice of initial seed voxel. *Left*: Reference full model simulation profile at the *t*_0_+30day time point. The *green box* highlights the region depicted in the panels on the right. *Right*: Tumor profiles produced by shifting the seed voxel anteriorly (*top*), rightward (*right*), posteriorly (*bottom*), or leftward (*left*). The seed voxel used for each profile is depicted in *white*, and the original seed voxel is depicted in *black* for comparison; these overlap in the cropped version of the reference image (*center*)

With regards to the robustness of the tumor profiles in relation to the placement of the seed cells, we illustrate in Fig. 3 the result of choosing seed voxels adjoining the one used to establish the preceding results. For this comparison, we utilized the full model, accounting for anisotropic diffusion and tumoral necrosis, with parameters *ρ*, *D*, and *η* fixed at the values chosen above, while simulating the profile at the *t*_0_+30day time point. We see that displacing the seed point rightward, leftward, anteriorly, or posteriorly produces qualitatively very similar, but perceivably different tumor profiles, as expected. Given that the *D* term effectively ensures that tumor cells have spread to adjacent voxels early in the simulation, these results are in keeping with our expectation that the model is not overly robust to the initialization. The mutual information metrics between the original profile (Fig. 3, center) and those produced by rightward, leftward, anterior, or posterior seed displacement were 4.135, 4.337, 4.019, and 4.448, respectively. When viewed in light of the values in Table 1, this quantitative comparison reinforces the notion that the model is relatively insensitive to small variations in seed placement.

## Discussion and conclusions

We have presented a model that generates tumor progression profiles that are qualitatively and quantitatively more representative of true GBM lesion growth, as compared to prior methods. The qualitative improvement was largely expected by construction, given that Eq. 3 represents the first GBM progression model to incorporate both anisotropic spread of tumor along fiber pathways as well as central necrosis. The quantitative analysis is interesting in that it reveals a progressive improvement in the accuracy of the simulated result as critical features are added to the model. In addition, the quantitative analysis suggests that for all models, the simulation accuracy decreases as the lesion progresses. This reinforces the intuitive notion that small or early GBM lesions are more morphologically similar and may even be well-represented by an isotropic growth model. As the tumor progresses, however, more complex features of its propagation become apparent, and these produce the main challenges for computational simulation, necessitating the use of more complex models.

There is significant potential clinical utility in this development of an accurate model for GBM progression that uniquely accounts for GBM necrosis. For example, the tumor necrosis rate has been shown to be inversely related to patient prognosis, with greater degrees of necrosis heralding worse clinical outcomes [15–17]. Others have presented evidence suggesting that the fatal tumor burden is related to the absolute number of tumor cells, a metric that clearly requires accurate modeling of central tumor cell death [5].

Beyond tumoral necrosis, the model parameters representing cell proliferation and diffusion may also have important clinical implications. We recall that, for the studied tumor, we estimated proliferation parameter *ρ*=0.33/day. However, a separate analysis of 32 GBM lesions revealed a mean *ρ* of 0.089/day (range 0.008–0.75), suggesting that the tumor we present in panels A and B of Fig. 2 exhibits a higher than average rate of cell division, a feature associated with poorer prognoses [5]. Furthermore, it has been shown that the relative values of *D* and *ρ* contribute to the accurate prediction of survival of GBM patients [3, 18, 19].

The primary limitation of the proposed technique is its reliance on the availability of serial imaging. Currently, standard treatment for GBM involves urgent resection and initiation of chemotherapy. Serial preoperative imaging is generally unavailable; the opportunity afforded by the patient in this report is a rare occurrence. Even so, the lack of a multidirectional diffusion-weighted sequence in our standard tumor imaging protocol has necessitated the use of an age and gender matched volunteer for modeling purposes; we would ideally establish these results in the same subject to eliminate all potential confounding effects of intersubject variability. Other similar efforts have reported the same challenge [18]. One potential compromise involves simulating tumor progression from a single imaging study using population average values for the model parameters; however, this sacrifices the advantages gained by the image-driven, tumor-specific formulation.

Serial post-operative imaging is widely available, but tumor progression in these images is almost invariably confounded by the effects of ongoing chemotherapy, radiation therapy, and repeat resections. Each of these greatly complicate the understanding of the progression of an individual cell population, which we provide the fundamental groundwork for in the present work. The most practically useful models will of course need to account for these ongoing treatment factors, one of which – radiation therapy – we have modeled separately elsewhere [24]. Specifically, we note that we have considered **D** to be time-invariant, and *ρ* and *η* to be constant across both time and space. As with all mathematical models, these assumptions represent simplifications of the underlying biological state, as tumor growth has the potential to alter the local cell diffusion tensor, and treatment, dedifferentiation, or tumor heterogeneity may alter the growth and necrosis parameters across space and time. It may further be necessary to incorporate information from additional modalities, such as MR spectroscopy and perfusion imaging, to account for heterogeneity in genotype and physiology within the tumor, especially as certain cell populations are differentially affected by treatment. Modeling GBM progression accurately over longer periods in patients actively undergoing treatment will thus certainly require further refinement of the model presented here to account for these characteristics.

Ultimately, we are also constrained by the limits of the underlying technologies, including the finite voxel size and the resulting inevitable cell population averaging, as well as the limits of diffusion tensor imaging in resolving complex and shallow-angle crossing geometries which may result in difficulties when applying our method to tumors in certain brain areas. We expect that improvements in these fundamental elements, such as those demonstrated by diffusion spectrum imaging and probabilistic tractography, will translate into more accurate tumor progression models [25].

In conclusion, given the heterogeneity of patient outcomes and response to therapy for GBM, we sought to construct an improved, lesion-specific model of tumor progression. In this report, we have developed a novel model with parameters that are driven by the post-contrast T_1_-weighted, T_2_-weighted, and diffusion-weighted imaging characteristics of each individual tumor. Finally, we have demonstrated both qualitatively and quantitatively that this model describes observed GBM progression, including central necrosis, more accurately than existing methods, with associated implications for clinical prognostic and therapeutic utility.

## Abbreviations

GBM: Glioblastoma multiforme
MI: Mutual information
MR: Magnetic resonance
MRI: Magnetic resonance imaging
